# Occurrence of retained placenta is preceded by an inflammatory state and alterations of energy metabolism in transition dairy cows

**DOI:** 10.1186/s40104-016-0085-9

**Published:** 2016-04-26

**Authors:** Elda Dervishi, Guanshi Zhang, Dagnachew Hailemariam, Suzana M. Dunn, Burim N. Ametaj

**Affiliations:** Department of Agricultural Food, and Nutritional Science, University of Alberta, Edmonton, AB T6G 2P5 Canada

**Keywords:** Innate immunity, Retained placenta, Serum metabolites, Transition dairy cows

## Abstract

**Background:**

Failure to expel fetal membranes within 24 h of calving is a pathological condition defined as retained placenta (RP). The objective of this investigation was to evaluate whether there are alterations in several selected serum variables related to innate immunity and carbohydrate and lipid metabolism that precede occurrence of RP in transition Holstein dairy cows.

**Methods:**

One hundred multiparous Holstein dairy cows were involved in the study. Blood samples were collected from the coccygeal vein during the −8 to +4 wks around parturition, once per week before the morning feeding. Six healthy control cows (CON) and 6 cows with RP were selected and serum samples at −8, −4, time of diagnosis of disease, and +4 wks relative to parturition were used for analyses. All samples were analyzed for lactate, non-esterified fatty acids (NEFA), β-hydroxybutyrate (BHBA), interleukin-1 (IL-1), interleukin-6 (IL-6), tumor necrosis factor (TNF), haptoglobin (Hp), and serum amyloid A (SAA).

**Results:**

Cows with RP had greater concentrations of serum lactate, IL-1, IL-6, TNF, and SAA in comparison with CON cows. Intriguingly, elevated concentrations of all five variables were observed at −8 and −4 wks before the occurrence of RP compared to healthy cows. Cows with RP also had lower DMI and milk production vs CON animals; however milk composition was not affected by RP.

**Conclusions:**

Cows with RP showed an activated innate immunity 8 wks prior to diagnosis of disease. Overall results suggest that serum IL-1, IL- 6, and TNF, and lactate can be used as screening biomarkers to indicate cows that might have health issues during the transition period.

## Background

It is widely accepted that transition period in dairy cows is characterized by a high incidence of metabolic and infectious diseases. Failure to expel fetal membranes within 24 h of calving is a pathological condition defined as retained placenta (RP) [1]. Normally, expulsion of placenta occurs 3–8 h after calf delivery [2]. An important sign associated with RP is degradation, discoloration, and hanging of fetid fetal membranes from the vulva > 24 h after parturition.

During the years four main hypotheses have been proposed with regards to retain placenta: uterine atony, edema of the chorionic villi, inflammatory states, and neutrophil inactivation [3, 4]. The later hypothesis was supported by data generated by Kimura et al. [1] that proposed a decrease in neutrophil functions before parturition. Later, LeBlanc [5] suggested that RP, metritis, and endometritis are diseases of immune function in the transition period, which begins at least 2 wks prepartum. Furthermore, Ametaj et al. [3] proposed that endotoxin might be involved in all four conditions by lowering uterine atony, inducing edema of the chorionic villi, initiating the inflammatory state, and causing neutrophilia from inability of neutrophils to move to inflammatory tissues.

There is an increasing body of evidence to support utilization of blood metabolites, cytokines, and acute phase proteins (APPs) as biomarkers of disease in dairy cattle. The use of APPs as biomarkers of cattle diseases also has been recently discussed by Bertoni and Trevisi [6]. In fact, several studies have reported alterations in blood metabolites, cytokines, and APPs in cows with RP. For example, concentrations of beta-hydroxybutyrate (BHBA) in the plasma of cows with RP induced by treatment with LPS, peaked at 14 d postpartum reaching sub-ketotic levels [7]. In addition, both prepartum non-esterified fatty acids (NEFA) and postpartum BHBA were associated with clinical diseases in dairy cows including RP [8, 9]. Moreover, Seifi et al. [10] showed greater concentrations of NEFA and BHBA in cows with RP. Also it has been suggested that elevated NEFA and ketone bodies are metabolic indicators of increased risk for RP [8, 11].

The decline in immune status around parturition and the incidence of RP (averages 5–15 %) in dairy cows, negatively affect animal welfare, herd health, fertility, and milk production. Retained placenta results in increased days open, calving to first heat interval, services per conception, and days from calving to first service, instigating important financial loses to dairy industry [1, 12, 13]. Because RP increases the odds of developing metritis and infertility [14, 15], mastitis, and lowers milk production [16] this disorder is considered an economically important issue for the dairy industry with estimated cost of $285 per case with an average incidence rate of 7.8 % [17].

The etiopathology of RP is not known and it will be of great interest to study the potential contributing factors that influence the occurrence of RP. Therefore, the objectives of this investigation were to evaluate whether there are alterations in selected blood variables related to innate immunity and carbohydrate and lipid metabolism that precede occurrence of RP, during the dry period starting at −8 wks prior to the expected day of parturition and up to +8 wks after calving and that might serve as screening biomarkers of RP, identification of cows at increased risk of developing RP, and learning more about the pathobiology of disease.

## Methods

### Animals and diets

One hundred pregnant Holstein dairy cows at the Dairy Research and Technology Centre, University of Alberta (Edmonton, AB, Canada), were used in a longitudinal study. Six pregnant multiparous Holstein dairy cows that were diagnosed with RP and six healthy control cows (CON) that were similar in parity, age, and body condition were selected for this nested case-control study (the mean parity of CON group is 3.2 ± 0.3 and the mean parity of RP group is 3.1 ± 0.3) age, and body condition score (BCS; the mean BCS for both groups is 3.17). There were 9 cows diagnosed with RP out of 100 cows sampled. Cows affected concurrently by RP and another disease were excluded from the study in order to better characterize RP. Out of 100 cows sampled only 6 were affected only by RP. All experimental procedures were approved by the University of Alberta Animal Policy and Welfare Committee for Livestock and animals were cared for in accordance with the guidelines of the Canadian Council on Animal Care [18].

The experimental period lasted for 17 wks starting from −8 wks before parturition to +8 wks postpartum (i.e., −8 wks to +8 wks, 0 wk means the week of calving) for each cow. Cows were housed in individual tie stalls bedded with sawdust and with free access to water throughout the experiment. One wk before the expected day of calving cows were transferred to the maternity barn and returned to their stalls on the following day of parturition. Diets were offered as TMR for ad libitum intake once daily at 0800 h to allow approximately 5 % orts. All TMR were formulated to meet or exceed the nutrient requirements of dry and early 680 kg lactating cows as per National Research Council guidelines [19]. Individual dry matter intake (DMI) was recorded daily throughout the 17 wks period by calculating the difference between the total daily diets given to each cow with the orts on the next morning. Since the onset day of lactation, cows were milked in their stalls twice per d at 0500 and 1600 h, and individual milk yield (MY) was recorded electronically. Milk samples collected on d 0, 14, 21, 35, and 49 relative to parturition (d 0 means the day of calving) were used for analysis of milk composition. Milk composition including crude protein (CP), milk fat, lactose, somatic cell count (SCC), milk urea nitrogen (MUN), and total solids (TS) were analyzed by mid-infrared spectroscopy (MilkoScan 605; A/S Foss Electric, Hillerød, Denmark) at the DHI Central Milk Testing Laboratory in Edmonton, Alberta.

### Monitoring of clinical health status

Health status (HS) of cows was monitored daily based on clinical signs of disease by trained individuals and on a weekly basis by a veterinary practitioner. All periparturient diseases and veterinary treatments were recorded for each cow throughout the entire experimental period. Diagnosis of pregnancy was performed routinely by a veterinary practitioner at 60–70 d post-insemination. Based on the artificial insemination (AI) data, supported with the information of pregnancy diagnosis, the expected date of parturition was estimated by adding 280 d from the day of AI. All cows were monitored daily starting at −8 wks prior to the expected date of calving and continuing up to +8 wks postpartum. The various external symptoms observed were - rectal temperature, ease of calving, body condition score (BCS), body temperature, and vaginal discharges (color and consistency).

In this study, RP was diagnosed if cows failed to expel fetal membranes within 24 h of calving. According to the veterinary protocol, cows with RP were treated with Tetrabol (Vetoquinol N.-A. Inc., lavatrie, Quebec, Canada) in the uterine cavity.

### Sample collection

Blood samples were obtained from the coccygeal vein once per wk at 0700 before feeding from −8 wks before parturition to +8 wks postpartum. All blood samples were collected into 10-mL vacutainer tubes (Becton Dickinson, Franklin Lakes, NJ, USA) and allowed to clot and kept at 4 °C until separation of serum. Clotted blood was centrifuged at 2,090 x *g* at 4 °C for 20 min (Rotanta 460 R centrifuge, Hettich Zentrifugan, Tuttlingen, Germany). The separated serum was aspirated from the supernatant gradually by transfer pipets (Fisher Scientific, Toronto, ON, Canada) without disturbing the sediment. The separated serum was transferred to a sterile 10-mL plastic test tube (Fisher Scientific, Toronto, ON, Canada). All serum samples were stored at −80 °C until analysis to avoid loss of bioactivity and contamination and were thawed on ice for approximately 2 h before use.

### Sample analyses

Serum metabolites. Quantitative determination of serum lactate, BHBA, and NEFA were measured by an enzymatic colorimetric method using commercially available kits provided by Sigma (St. Louis, MO, USA), Stanbio Laboratory (Boerne, TX, USA) and Wako Chemicals (Richmond, VA, USA), respectively. The detailed methods have been described previously by Ametaj et al. [20]. Briefly, according to the manufacturers’ instructions, the lower detection limits of the kits were 0.06 mg/dL, 0.125 μmol/L, and 0.50 μEq/L, respectively. The principle of the lactate assay involves reduction in the colorless tetrazolium salt by an NADH-coupled enzymatic reaction to formazan, which develops a red color change proportional to the lactate concentration. BHBA test involved the basic principle of conversion of serum BHBA to acetoacetate and NADH by BHBA dehydrogenase in presence of NAD. Then, the NADH reacts with 2-p-iodophenyl-3-p-nitrophenyl-5-phenyltetrazolium chloride (INT) in the presence of diaphorase to form a pink colored adduct proportional to the concentration of BHBA in the serum. The principle of NEFA kit involved acylation of coenzyme A (CoA) by fatty acids in the serum in presence of acyl-CoA synthetase and production of hydrogen peroxide in presence of acyl-CoA oxidase. Hydrogen peroxide, together with peroxidase, permits the oxidative condensation of 3-methyl-N-ethyl-N-β-hydroxy ethyl-O-aniline with 4-aminoantipyrine to produce a purple color change, which is proportional to the serum NEFA concentrations. All samples were tested in duplicate and absorbance of standards and samples vs a blank for lactate, BHBA, and NEFA were read at 492, 505, and 550 nm, respectively, in a microplate reader (Spectramax 190, Molecular Devices Corporation, Sunnyvale, CA, USA). The intra-assay variation of all the three assays was controlled by coefficient of variation (CV) limits < 10 %.

Serum cytokines: Concentration of IL-1 in the serum was assayed by a commercially available bovine ELISA kit (Cusabio Biotech Co. Ltd., Wuhan, China) with mAb (monoclonal antibodies) specific for IL-1 coated on the walls of the microplate strips provided. The procedure involved the basic principle of a competitive inhibition enzyme immunoassay between biotin-conjugated IL-1 and IL-1 with the pre-coated antibody. All samples (50 μL) were tested in duplicate in microtitration wells with biotin-conjugated IL-1 according to the manufacturer’s instructions. The plates were washed with wash buffer after the incubation for 60 min at 37 °C, followed by addition of 50 μL of horseradish peroxidase (HRP)-avidin. Samples were incubated for 30 min at 37 °C. Then, they were washed 3 times with buffer, and 50 μL substrate A and 50 μL of substrate B reagent were added to each well. After incubation at 37 °C for 15 min, the resulting color reaction was read at 450 nm by a microplate reader (Spectramax 190, Molecular Devices Corporation, Sunnyvale, CA, USA) within 10 min, and the final IL-1 concentration was calculated using a 4-parameter logistic curve fit. The sensitivity of this assay was 250 pg/mL, and the intra-assay CV was < 10 %.

Concentration of IL-6 in the serum was measured with a bovine ELISA kit provided by Uscnk Life Science Inc. (Wuhan, China) as described by the manufacturer. The detection limit of the assay was 7.8 pg/mL and the intra-assay variation of all IL-6 assays was controlled by CV limits < 10 %. The principle of the IL-6 test involves a sandwich enzyme immunoassay, which exhibits a yellow color change proportional to IL-6 concentration. Samples or standards were added to the microtiter plate wells with a biotin-conjugated antibody specific for IL-6 with all samples in duplicate. Then, HRP-avidin was added and the plate was incubated for 90 min at 37 °C in total. After 3,3,5,5-tetramethylbenzidine (TMB) substrate and sulphuric acid solution were added, the color change was measured spectrophotometrically at a wavelength of 450 nm (Spectramax 190, Molecular Devices Corporation, Sunnyvale, CA, USA).

Concentration of TNF in the serum was determined by a commercially available bovine ELISA kit (Bethyl Laboratories, Inc., Montgomery, TX, USA) using a method described previously by Iqbal et al. [21]. Briefly, all samples were tested in duplicate and the optical density values were read at 450 nm on a microplate spectrophotometer (Spectramax 190, Molecular Devices Corporation, Sunnyvale, CA, USA). The detection range of TNF assay was between 0.078 and 5 ng/mL, and the intra-assay CV was lower than 10 %.

Serum APPs: Methods used for the measurement of concentration of Hp (Tridelta Development Ltd., Co.Kildare, Ireland) and SAA (Tridelta Development Ltd.) in the serum were described previously in detail by Iqbal et al. [22]. Briefly, serum samples for Hp were not diluted. The minimum detection limits for Hp, and SAA assays were 2.5 mg/mL, and 18.8 ng/mL respectively. All samples were tested in duplicate and the optical densities were measured at 600 nm for Hp and 450 nm for SAA. The intra-assay variations of APPs assays were controlled by the CV limits at no more than 10 % and for those greater than 10 % samples were reanalyzed.

### Statistical analyses

To perform standard longitudinal study comparisons between the two groups, the group of healthy cows with those of RP one were compared at each time point (−8, −4, disease diagnosis, and +4 wks).

For parametric analysis of the data ANOVA was used by MIXED procedure of SAS (SAS Institute Inc., Cary, NC, USA, Version 9.2) according to the following model:

$$ {\mathrm{Y}}_{\mathrm{i}\mathrm{j}\mathrm{k}}=\upmu +{\mathrm{S}}_{\mathrm{i}}+{\mathrm{W}}_{\mathrm{j}}+{\left(\mathrm{S}\mathrm{W}\right)}_{\mathrm{i}\mathrm{j}}+{\mathrm{e}}_{\mathrm{i}\mathrm{j}\mathrm{k}} $$

where Y_ijk_ is the observations for dependent variables, μ represents the population mean, S_i_ is the fixed effect of health status i (i = 1-2, sick cows compared with healthy control separately), W_j_ is the fixed effect of measurement week j (j = 1–4 or 1 –17), SW_ij_ is the fixed effect of health status by week interaction, and e_ijk_ is the residual error.

Measurements taken at different weeks on the same cow were considered as repeated measures in the ANOVA. The variance–covariance structure of the repeated measures was modeled separately for each response variable according to the lowest values of the fit statistics based on the Bayesian Information Criteria, and an appropriate structure was fitted. Degrees of freedom were approximated by the method of Kenward-Roger (ddfm = kr).

In order to identify early indicators of RP, average serum concentrations in the week of diagnosis, as well as at −8 and −4 wks before the expected day of parturition were compared using *t*-test of SAS 9.2 between healthy cows and cows with RP. Data are exhibited as least-squares means (LSM) and the respective standard error of the mean (SEM). All statistical tests were two-sided. Significance was declared at *P* < 0.05, and tendency was defined at 0.05 < *P* < 0.10.

Multivariate analysis was performed using MetaboAnalyst [23]. Recommended statistical procedures for principal component analysis (PCA) and partial least squares discriminant analysis (PLS-DA) were followed according to previously published protocols [23]. To perform a longitudinal study combined with cross-sectional comparisons between two groups, we compared the group of CON cows with that of RP one at each time point ( −8, −4, disease diagnosis, and +4 wks). In the PLS-DA model, a variable importance in the projection (VIP) plot was used to rank the variables based on their importance in discriminating RP group from the CON group of cows. Variables with the highest VIP values are the most powerful group discriminators. Typically, VIP values > 1 are significant and VIP values > 2 are highly significant. Biomarker profiles and the quality of the biomarker sets were determined using receiver-operator characteristic (ROC) curves as calculated by MetaboAnalyst 3.0 [24]. Paired sensitivity and false-positive ratios (1-specificity) at different classification decision boundaries were calculated. A ROC curve is plotted with sensitivity values on the Y-axis and the corresponding false-positive rates (1-specificity) on the X-axis. ROC curves are often summarized into a single metric known as the area under the curve (AUC), which indicates the accuracy of a test for correctly distinguishing one group such as RP cows from CON ones. If all positive samples are ranked before negative ones, the AUC is 1.0, which indicates a perfectly discriminating test. The 95 % confidence interval (CI) and P values were calculated. A rough guide for assessing the utility of a biomarker set based on its AUC is 0.9 ~ 1.0 = excellent; 0.8 ~ 0.9 = good; 0.7 ~ 0.8 = fair; 0.6 ~ 0.7 = poor; 0.5 ~ 0.6 = fail.

## Results

### Serum metabolites

Concentrations of metabolites for the prepartum period are shown in Tables 1 and 2. Overall data demonstrated that concentration of lactate in cows with RP were greater compared to CON ones (4,502 vs 2,258 μmol/L; *P* < 0.05). Furthermore, comparison of means at −8 wk before parturition showed that cows with RP tended to have greater concentrations of lactate compared to those of CON (*P* = 0.05), reaching significance at −4 wk before parturition (*P* < 0.05) and remaining elevated until +4 wks after parturition (*P* < 0.05). No differences between the two groups of cows were detected with regards to concentrations of NEFA and BHBA in the serum; however, data showed an effect of week of sampling on those variables (*P* < 0.05).

**Table 1:** Data of dry matter intake, milk production, milk composition, and selected metabolites, cytokines, and APP in the serum of dairy cows with (*n* = 6) and without (*n* = 6) retained placenta (RP) during the periparturient period

	Group^a^			Effect,^b^ *P*-value		
Item	CON	RP	SEM	Hs	Wk	Hs × Wk
DMI^c^, kg/d	18.64	16.45	0.56	0.02	<0.01	0.23
Milk production^d^, kg/d	42.37	32.58	2.80	0.02	<0.01	0.07
Temperature, °C	38.43	38.42	0.05	0.88	<0.01	0.63
BCS	2.93	3.08	0.11	0.39	<0.01	074
Milk composition ^e^, %, unless otherwise stated						
Fat	3.84	3.80	0.22	0.91	0.33	0.28
Protein	2.88	2.92	0.10	0.77	<0.01	0.15
Fat:protein ratio	1.36	1.28	0.09	0.57	0.21	0.26
Lactose	4.56	4.49	0.04	0.35	0.04	0.49
SCC, 10^3^ cells/mL	30.83	50.79	4.2	0.01	0.22	0.30
Milk urea N, mg/dL	15.56	12.85	0.98	0.11	0.25	0.10
TS	12.21	13.65	0.68	0.22	0.29	0.56
Serum parameters ^f^						
Lactate, μmol/L	2,258	4502	456	0.01	0.27	0.36
NEFA, mmol/L	397.13	275.87	73.11	0.30	<0.01	0.50
BHBA, μmol/L	595.81	431.32	67.51	0.13	<0.01	0.14
IL-1, pg/mL	296.64	313.39	3.69	0.02	<0.01	0.53
IL-6, pg/mL	26.74	57.71	12.24	0.20	<0.01	<0.01
TNF-α, ng/mL	0.19	1.22	0.11	<0.01	0.42	0.68
Haptoglobin, mg/mL	0.15	0.36	0.07	0.10	0.16	0.10
SAA, ug/mL	8,477	23,165	2,311	<0.01	0.81	0.95

^a^CON = cows without retained placenta (health control); RP = cows with retained placenta

^b^Effect of health status (Hs), sampling week (Wk), and health status by sampling week interaction (Hs × Wk)

^c^Dry matter intake was calculated from week −8 to +8 relative to parturition

^d^Milk production was calculated from week +1 to +8 relative to parturition

^e^Milk compositions were determined on week +2, +3, +5, +7 relative to parturition

^f^Serum parameters were calculated from week −8, −4, disease and +4 relative to parturition

**Table 2:** Data of dry matter intake, milk production, milk composition and serum variables at the diagnosis week, and concentrations of serum indicators prior to the diagnosis of retained placenta (RP)

	−8 wks before parturation			−4 wks before parturation			RP diagnosis week^a^			+4 wks after parturition		
Item	CON	RP	*P*-value	CON	RP	*P*-value	CON	RP	*P*-value	CON	RP	*P*-value
DMI, kg/d	16.27 ± 0.82	12.48 ± 0.88	0.02	15.92 ± 0.10	14.28 ± 0.78	0.08	20.34 ± 0.56	15.55 ± 0.88	<0.01	21.68 ± 0.68	20.71 ± 1.34	0.53
Milk production, kg/d							30.95 ± 0.32	24.61 ± 3.52	0.13	42.04 ± 2.21	38.32 ± 4.23	0.45
Temperature, °C	38.23 ± 0.15	37.90 ± 0.30	0.42	38.45 ± 0.08	38.42 ± 0.11	0.81	38.42 ± 0.10	38.77 ± 0.16	0.09	38.32 ± 0.05	38.34 ± 0.13	0.87
BCS	2.75 ± 0.22	3.13 ± 0.22	0.18	3.04 ± 0.16	3.21 ± 0.10	0.41	3.17 ± 0.17	3.17 ± 0.15	1.0	2.58 ± 0.08	2.75 ± 0.14	0.41
Milk composition, %, unless otherwise stated												
Fat							5.08 ± 0.45	4.29 ± 0.44	0.25			
Protein							3.00 ± 0.10	3.12 ± 0.13	0.50			
Fat : Protein ratio							1.69 ± 0.12	1.38 ± 0.14	0.13			
Lactose							4.54 ± 0.05	4.38 ± 0.05	0.05			
SCC, 10^3^ cells/mL							28.33 ± 5.63	108.67 ± 39.46	0.10			
Milk urea N, mg/dL							15.39 ± 0.76	13.33 ± 1.66	0.29			
TS							12.21 ± 0.31	13.98 ± 1.14	0.19			
Serum parameters												
Lactate, μmol/L	2,455 ± 348	4,855 ± 692	0.05	2,162 ± 184	5,507 ± 933	0.03	2,227 ± 320	4,458 ± 925	0.05	2,100 ± 129	3,726 ± 468	0.04
NEFA, mmol/L	140.79 ± 32.77	193.32 ± 54.25	0.41	194 ± 47	182 ± 37.61	0.87	756.51 ± 232.01	471 ± 146	0.34	500.43 ± 151.71	269.48 ± 69.13	0.23
BHBA, μmol/L	352 ± 37.71	340 ± 63.99	0.87	312 ± 18	366 ± 49.62	0.30	827 ± 151.50	553 ± 85	0.15	896.73 ± 188.3	553.15 ± 85.24	0.13
IL-1, pg/mL	317 ± 6.04	347 ± 15.90	0.04	321 ± 1.59	337. ± 6.04	0.02	277 ± 5.42	290 ± 7.36	0.05	270.57 ± 2.87	287.51 ± 8.99	0.05
IL-6, pg/mL	19.23 ± 5.67	100 ± 41.45	0.02	48.24 ± 17.51	69.39 ± 18.43	0.34	23.17 ± 5.18	35.65 ± 21.63	0.59	15.71 ± 3.27	32.99 ± 7.32	0.10
TNF-α, ng/mL	0.34 ± 0.03	1.26 ± 0.30	0.03	0.27 ± 0.05	1.31 ± 0.22	<0.01	0.06 ± 0.03	1.37 ± 0.41	0.01	0.07 ± 0.04	1.04 ± 0.25	0.05
Haptoglobin, mg/mL	0.19 ± 0.03	0.34 ± 0.25	0.58	0.15 ± 0.01	0.07 ± 0.02	<0.01	0.12 ± 0.01	1.06 ± 0.31	<0.01	0.16 ± 0.01	0.17 ± 0.17	0.98
SAA, ug/mL	8,447 ± 3373	24,584 ± 7,794	0.04	3,461 ± 342	19,378 ± 6,445	0.03	10,401 ± 1,722	21,706 ± 8,746	0.04	11,797 ± 1,853	23,955 ± 10,369	<0.01

^a^Cows were diagnosed with retained placenta (*n* = 6) ranging from week 0 to +1. CON = cows without retained placenta (health control); RP = cows with retained placenta

### Serum cytokines

Data with respects to concentrations of IL-1, IL-6, and TNF in the serum showed that cows with RP had greater concentrations of IL-1 and TNF throughout the study (*P* < 0.05); with sampling week also having an effect on the results of IL-1 (Table 1 and 2). Comparisons of means also showed greater concentrations of IL-1 and TNF in cows with RP (*P* < 0.05) at −8 and −4 wks prior to parturition. Concentrations of IL-1 and TNF tended to be greater in cows with RP at +4 wks after parturition. Moreover, at −8 wks, cows with RP had greater concentrations of IL-6 compared to healthy cows. Elevated concentrations of IL-6 were not maintained at −4 wks before parturition, and at the week of diagnosis of RP. Data showed an effect of week of sampling and interaction of health status and week of sampling on IL- 6 (*P* < 0.05).

### Serum acute phase proteins

Results with regards to concentrations of Hp and SAA in the serum are shown in Table 1 and 2. Concentrations of Hp in the serum of cows with RP were lower compared to healthy cows at −4 wks before parturition (*P* < 0.05), however, during the week of diagnosis of RP concentrations of Hp in cows with RP increased almost 10-fold compared to healthy cows (*P* < 0.01). Cows with RP had greater concentrations of SAA compared to CON ones throughout the study (*P* < 0.05). Interestingly concentrations of SAA in RP cows were greater starting from wk −8 and −4 prior to parturition (*P* < 0.01) and remained elevated until +4 wks after calving (P < 0.05).

### Dry matter intake, milk production, and milk composition

Changes in DMI, concentrations of milk fat, protein, and fat:protein ratio in the milk are shown in Table 1 and 2. Moreover, the number of somatic cell count (SCC), milk urea N (MUN), total solid (TS), and lactose in the milk are shown in Table 1 and 2. Overall DMI in cows with RP was lower compared to controls (*P* < 0.05). The sampling week also had an effect (*P* < 0.05), indicating wk-to-wk variation in DMI. Comparison of means demonstrated that DMI was lower at −8 wks before parturition (*P* = 0.05) and during the week of diagnosis of RP (*P* < 0.01). At −4 wks before calving DMI tended to be lower in cows with RP (*P* = 0.08).

Moreover, cows with RP showed lower milk production throughout the experimental period and during the week of diagnosis of RP (32.58 vs 42.37 kg/d; *P* < 0.05). In addition, SCC were greater in cows with RP compared to healthy controls during the study (*P* < 0.05), but not during the week of diagnosis of RP.

The amount of lactose in the milk of cows with RP tended to be lower only at diagnosis week (*P* = 0.05), whereas protein, fat:protein ratio, MUN, and TS did not show differences between RP and healthy controls during the postpartum weeks of the study (*P* > 0.05).

### Results of multivariate analyses

**Fig. 1:**
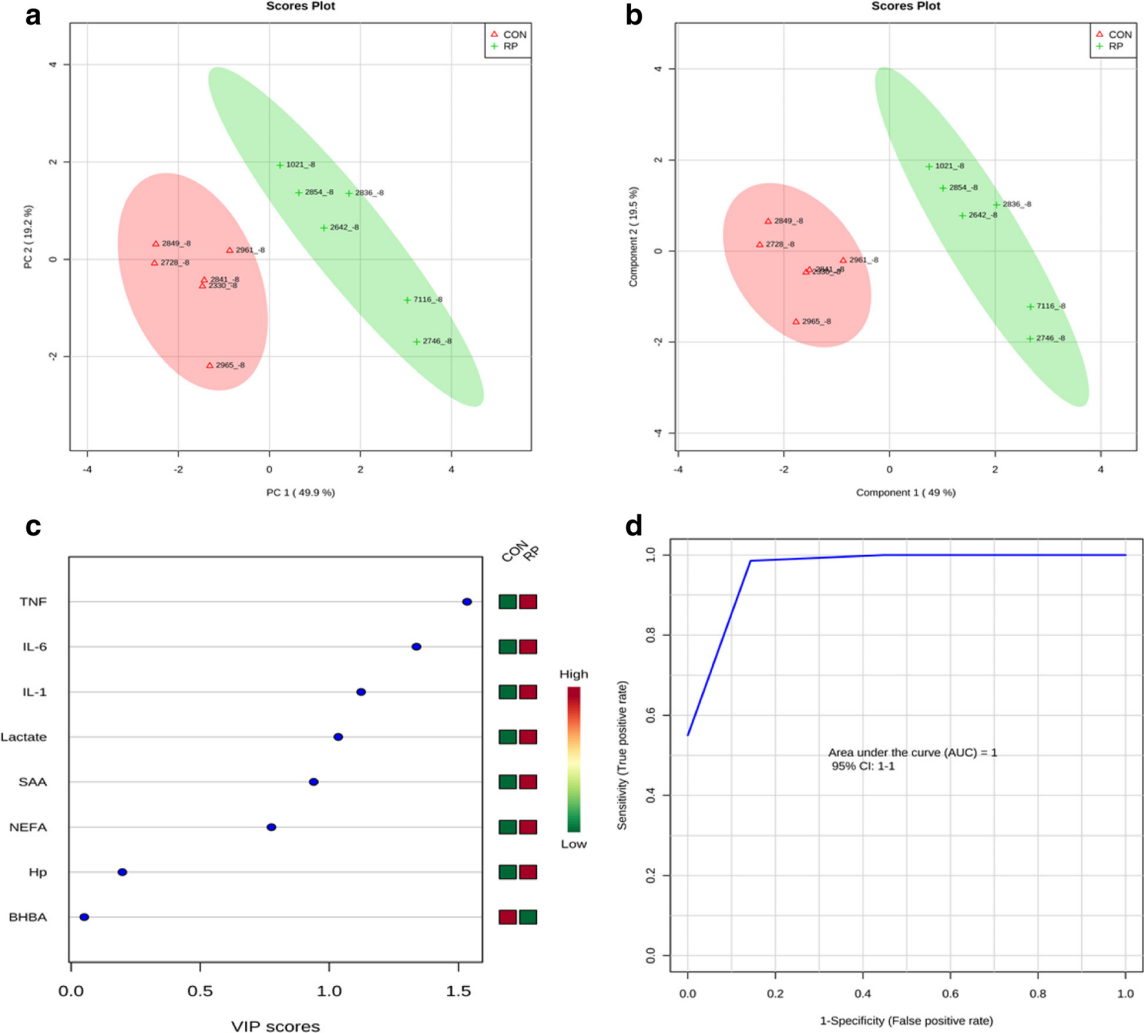
**a** Principal component analysis (PCA) and (**b**) Partial least squares-discriminant analysis (PLS-DA) of 6 control and 6 RP cows at −8 wk before parturition showing 2 separated clusters for 2 groups. **c** Variables ranked by variable importance in projection (VIP), and (**d**) Receiver-operator characteristic (ROC) curve of 6 CON and 6 RP cows at −8 wks before parturition for the top 3 serum variables

**Fig. 2:**
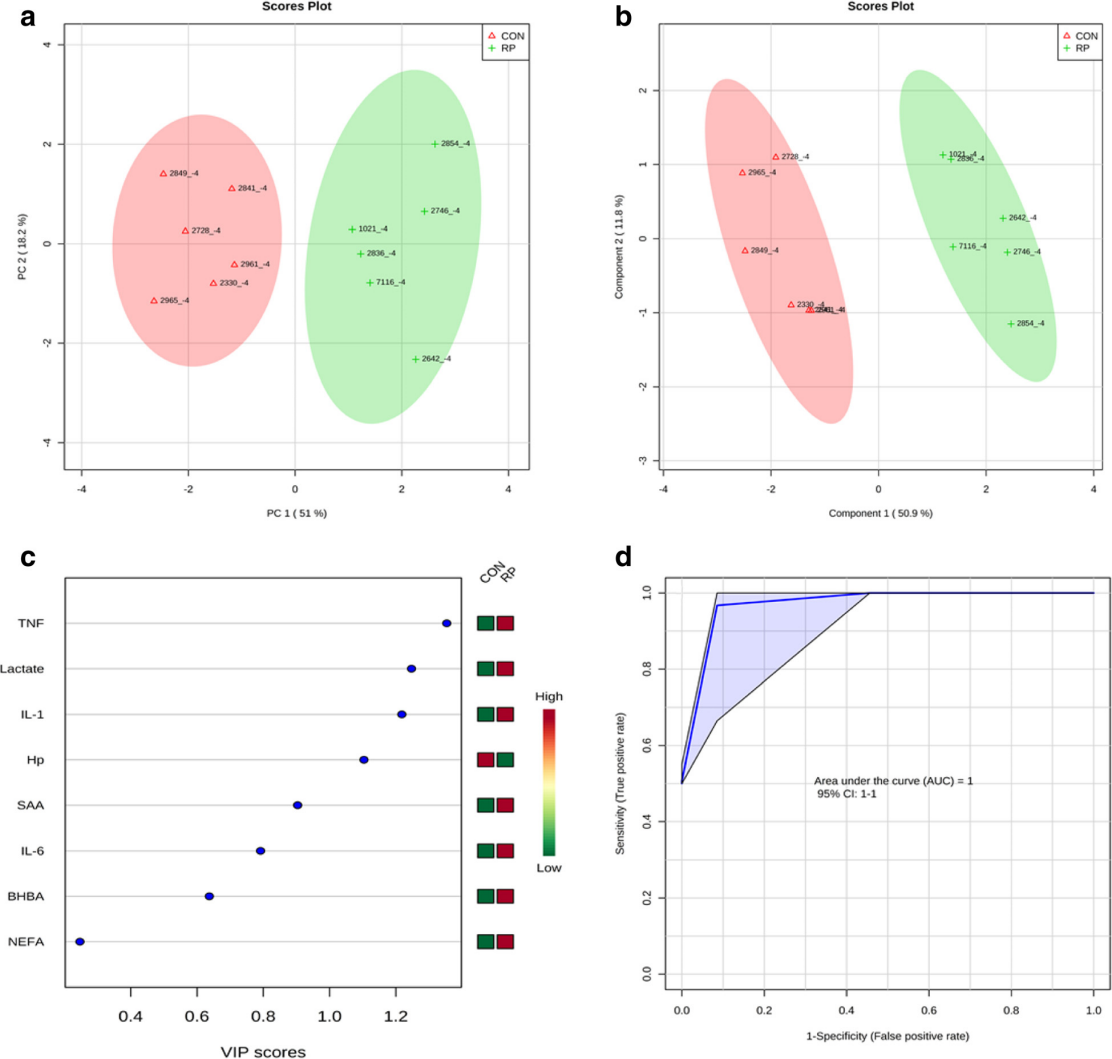
**a** PCA and (**b**) PLS-DA of 6 control and 6 RP cows at −4 wks before parturition showing 2 separated clusters for 2 groups. **c** VIP, and (**d**) ROC curve of 6 CON and 6 RP cows at −4 wks before parturition for the top 3 serum variables

**Fig. 3:**
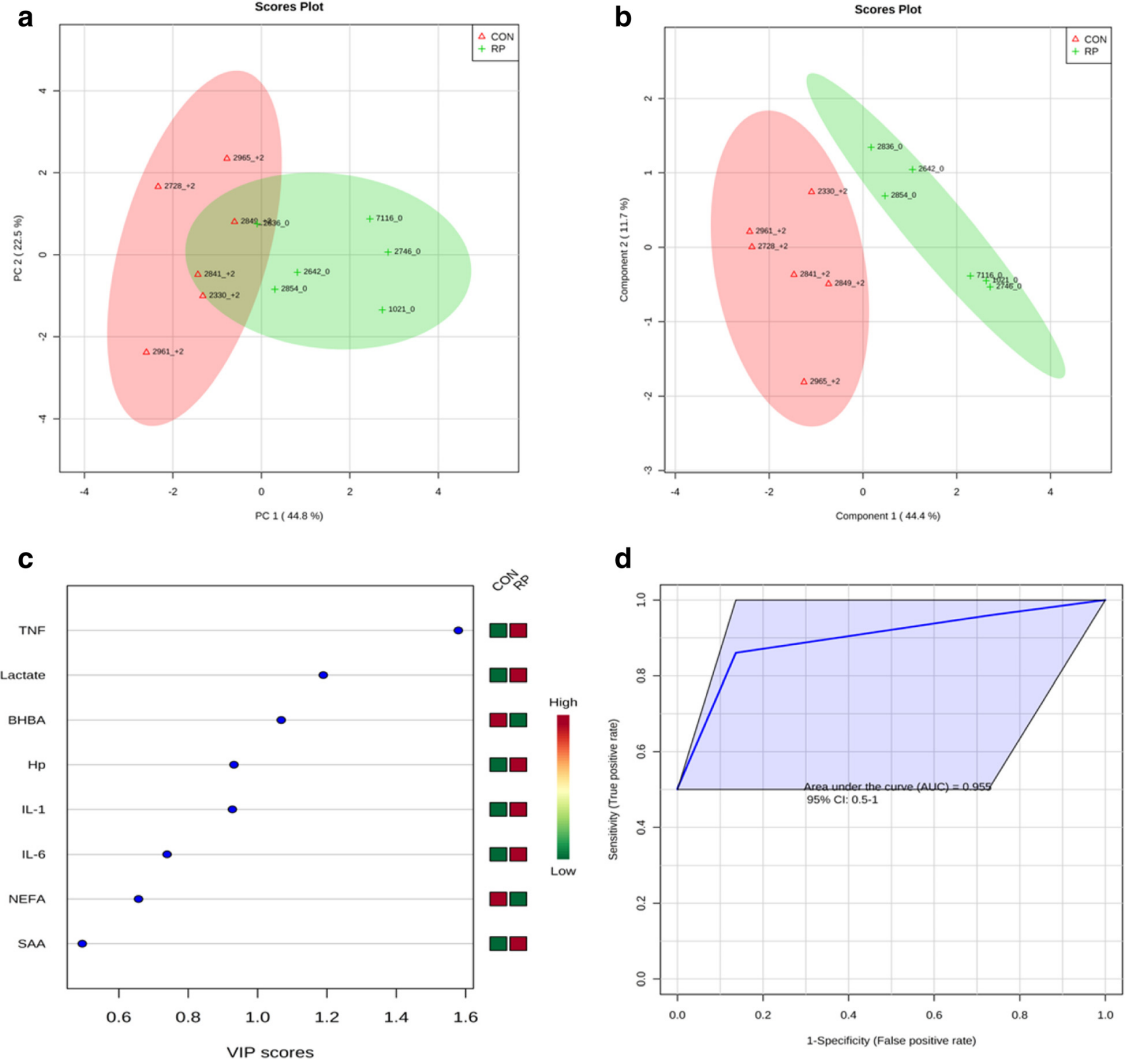
**a** PCA and (**b**) PLS-DA of 6 control and 6 RP cows at disease week showing 2 separated clusters for 2 groups. **c** VIP, and (**d**) ROC curve of 6 CON and 6 RP cows at disease wk for the top 3 serum variables

**Fig. 4:**
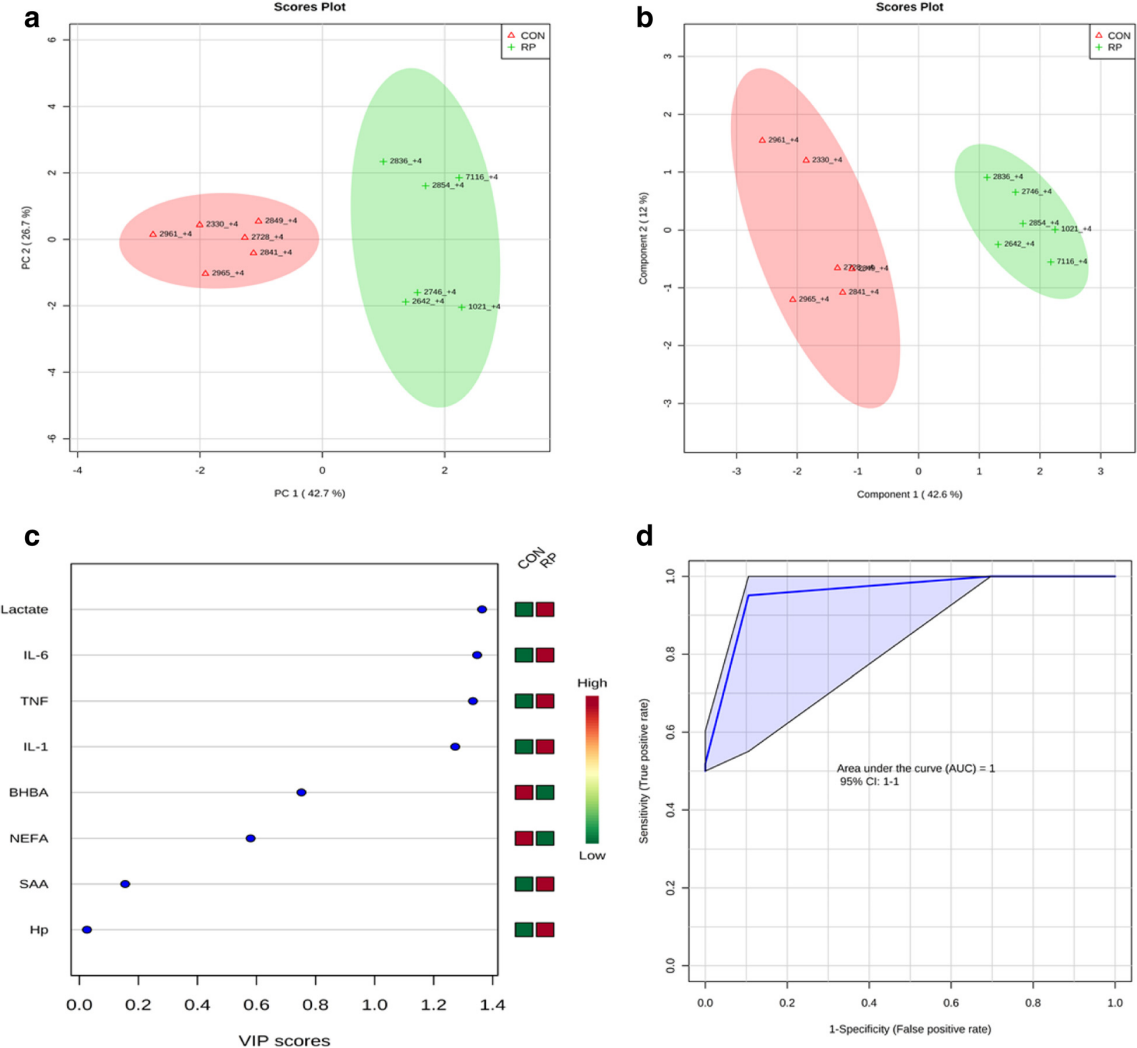
**a** PCA and (**b**) PLS-DA of 6 control and 6 RP at 4 wks after parturition showing 2 separated clusters for 2 groups. **c** VIP, and (**d**) ROC curve of 6 CON and 6 RP cows at +4 wks after parturition for the top 3 serum variables

Principal component analysis of healthy cows versus those with RP at −8 wks showed that the first 2 PC (principal components) covered 69.1 % of the observed variance in the sample set (Fig. 1a). In addition, PLS-DA scores plot revealed that it is possible to discriminate between cows that did not have RP and those that did at −8 wks before calving (Fig. 1b). When healthy cows and RP cows were compared at −4 wks prepartum (Fig. 2a), PCA analysis showed that the first 2 PC covered 69.2 % of the observed variance in the sample set. PLS-DA scores plot revealed that it is possible to discriminate between healthy cows and those with RP at −4 wks before parturition (Fig. 2b). Similar results were obtained during the week when RP was diagnosed (Fig. 3a and Fig. 3b) and +4 wks postpartum (Fig. 4a). A VIP plot in a PLS-DA model at 4 time points in which the serum variables were ranked based on their contribution to discriminating the RP cows from CON ones are shown in Figs. 1c, 2c, 3c, and 4c. The VIP plots indicated that TNF, IL-6 and IL-1 at −8 wks; TNF, lactate, and IL-1 at −4 wks; TNF, lactate, and BHBA at the week of diagnosis of RP; and lactate, IL- 6, and TNF at +4 wks were the strongest discriminating variables for separating RP cases form CON cows. The heat map on the right side of the 4 VIP plots indicated that these variables were enhanced in cows with RP relative to CON cows.

A ROC curve plot indicating the performance of the top 3 metabolites in predicting which cows will develop RP at −8 and −4 wks using a PLS-DA model are shown in Figs. 1d and 2d. The AUC for the curve at −8 wks is 1 (95 % CI, 1-1), which indicates that TNF, IL-6, and IL-1 together at −8 wks have very strong predictive abilities. The AUC for the curve at −4 wks was 1 (95 % CI, 1-1), which suggest that at −4 wks the combination of TNF, lactate, and IL-1 have very strong predictive abilities. These results demonstrate that biomarker models developed at −8 and −4 wks could be used to predict which cows are susceptible to develop RP after parturition. The ROC curve developed based on three metabolites TNF, lactate, and BHBA at the week of diagnosis of RP indicates that this three-metabolite set were a highly significant biomarker for diagnosis of RP, with AUC, 0.955 (95 % CI, 0.5–1, Fig. 3d). Moreover, multivariate models (ROC curves) combining 3 discriminating variables lactate, IL- 6, and TNF at +4 wks produced an area under the receiver-operating curve of 1 (95 % CI: 1-1, Fig. 4d).

## Discussion

The objective of this investigation was to evaluate whether there were alterations in some of the most studied blood variables related to some aspects of innate immunity and carbohydrate and lipid metabolism in transition dairy cows that retained their fetal placental membranes more than 24 h after parturition. Indeed, the results of our study revealed that cows that developed RP had greater concentrations of serum IL-1, IL-6, TNF, SAA, and lactate in comparison with CON cows. Intriguingly, elevated concentrations of all five variables were observed at −8 and −4 wks before the occurrence of RP compared to CON cows. In addition, cows with RP also had lower DMI and milk production compared to CON animals.

Results of this study showed that concentrations of IL-1 and IL-6 were significantly greater in cows with RP throughout the study, starting at −8 wks before calving. Interleukin-1 is a central mediator of innate and adaptive immunity and inflammation. It is a cytokine produced mainly by activated macrophages in the liver, lymph nodes, udder, adipose tissue, systemic circulation or other inflamed tissues or organs and also plays a significant role in the activation of APR [25]. In the present study, the overall concentration of IL-6 was not different between healthy cows and those with RP; however, serum IL-6 was greater in RP cows at −8 wks before parturition. Interleukin-6 plays a significant role during the transition from innate to adaptive immunity. The reasons why both IL-1 and IL-6 were greater in would-be-RP cows suggest presence of an inflammatory insult starting at −8 wks before parturition. This assumption also is supported by concurrent elevated concentrations of TNF in the serum of RP cows, which has a critical role in initiating the host immune response by triggering the release of IL-1 and IL-6. Our results are in line with other studies that have reported that greater serum TNF, at approximately 15 d before parturition, is associated with greater risk of RP [26, 27]. Our data suggest that combination of serum TNF, IL-1, and IL-6 can be used to screen cows for the potential occurrence of RP starting at −8 wks prior to calving. This suggestion should be taken with caution because proinflammatory cytokines are global indicators of inflammation but not of a specific disease. To our best knowledge this is the first study to relate high concentrations of IL-1, IL-6, and TNF, at least 8 wks before parturition, with potential development of RP. It is speculated that the reason why serum IL-1, IL-6, and TNF are greater in RP cows might be presence of an inflammatory insult in the mammary gland at 8 wks before parturition. This is in agreement with the reported data that high concentrations of cytokines, or inflammatory indicators in cows during the dry period have been associated with greater incidence of mastitis or to inflammatory events around calving [28, 29]. However, additional research is warranted to clarify the reasons behind elevated concentrations of pro-inflammatory cytokines prior to occurrence of RP.

Pro-inflammatory cytokines like IL-1 and IL-6 are known to trigger the release of APPs from hepatocytes [30, 31]. Indeed, in the present study serum concentrations of SAA were greater in cows with RP, throughout the study, starting at −8 wks before calving and up to +4 wks after parturition. In addition, concentrations of Hp in cows with RP increased almost 10-fold compared to healthy cows at the week of diagnosis of RP. Serum amyloid A binds to high density lipoproteins and participates in expedited clearance of translocated endotoxin through the liver [32], whereas Hp binds hemoglobin and prevents utilization of iron by bacteria translocated into the blood circulation [33] and plays a significant role in neutrophil recruitment to the inflammation site. Numerous studies have shown that cows experiencing an APR around parturition have greater odds of developing metabolic diseases [32, 34, 35]. The results of this study support the idea that APR in cows with RP is probably due to the existence of an inflammatory state in cows starting at 8 wks before parturition. Ametaj et al. [3] proposed that LPS from *E. coli* might be involved in the incidence of RP. In addition, in a previous study we demonstrated that intermittent intravenous infusion of LPS in transition dairy cows increased the incidence rate of RP and was associated with greater systemic concentrations of SAA and Hp [7]. This study supports our assumption that an infection involving Gram-negative bacteria might be involved in triggering of APR and the release of positive APPs in the systemic circulation prior to and during occurrence of RP. To our best knowledge this is the first study to report that concentrations of SAA increase at least 8 wks before parturition in cows that retained their fetal membranes after calving.

Results of this study also showed that an inflammatory insult associated with an APR preceded RP in postpartum dairy cows. An intriguing question is whether activation of innate immunity during the dry off period contributes to retention of fetal membranes in the transition dairy cows? Although there are no published reports with respects to how an inflammatory condition might affect RP in dairy cows, research in human subjects indicates that inflammatory leukocytes of maternal origin migrate to placental membranes and cause chorioamnionitis (inflammation of fetal membranes on both maternal (chorion) and fetal sides (amnion) [36]. It is speculated that inflammatory leukocytes might infiltrate from the dam’s systemic circulation to the placenta and even fetus itself and trigger inflammation associated with RP and harmful effects on the fetus.

Another important finding of this study was that cows with RP had greater concentrations of lactate in the serum starting at −8 wks before parturition, at −4 wks, disease diagnosis wk, and even at +4 wks postpartum. The reason for greater lactate in the serum is not clear. It is speculated that lactate is coming from muscle tissue or mammary gland. Previous research has indicated that during endotoxemia there is increased blood lactate, originating from altered muscle metabolism [37]. Given the obseervation that would-be-RP cows had greater concentrations of cytokines and APPs in the serum during the dry off period, it is reasonable to speculate that translocation of endotoxin might have also occurred in those cows. Blood lactate has been previously associated with other metabolic disease like mastitis [38]. In addition, our team has reported that greater concentrations of lactate precede milk fever and laminitis in transition dairy cows [39, 40]. Intriguingly lactate has been shown to inhibit motility of T cells to the inflammation site and their effector functions [41]. In addition, lactate has suppressive effects on T cell cytotoxic activity [42], alters antigen-presenting ability of dendritic cells [43], and interferes with NK cell activity [44]. Lactate also has been demonstrated to affect the motility of neutrophils and their killing capabilities, and trigger anti-inflammatory responses by suppression of inflammasome and proinflammatory cytokine production.

The overall DMI in cows that retained their fetal membranes was lower compared to CON cows. Dry matter intake was lower in RP cows starting at −8 wks before parturition (16.27 ± 0.82 vs 12.48 ± 0.88 kg) and at the wk of diagnosis of RP. One of the most important physiological changes occurring during the dry off period is the decrease in feed intake [36, 45] and lower feed intake is known to be associated with a drop in body weight. This is in agreement with previously reported findings that proinflammatory cytokines affect both DMI and disease incidence around calving [28].

Retained placenta is associated with lowering of milk production and significant financial losses to the dairy industry [1]. In this study cows with RP had lower milk production throughout the postpartal experimental period, which is in line with previous reports including Lucey et al. [15] and Sartori et al. [46]. Overall, cows with RP produced 9.79 L of milk/d less compared to their healthy counterparts, which implies a loss of 2.74$/d/cow, without taking into consideration the cost of medications and veterinary services. The reason why cows with RP had lower milk production might be related to the increase in concentrations of circulatory TNF. Infection by Gram-negative bacteria and their cell wall component, lipopolysaccharide (LPS), has been shown to inhibit production of prolactin in the pituitary gland, mediated by TNF [47]. This assumption also is supported by a previous investigation demonstrating a decrease in milk yield in lactating cows after parenteral administration of TNF [48].

Furthermore the results of PCA and PLS-DA showed separation between healthy cows and those with RP starting at −8 wks before parturition. The metabolic and innate immunity alterations prior to calving suggest presence of an inflammatory insult prior to parturition. This substantiates the notion that alterations in blood plasma metabolites starts prior to the onset of the clinical signs of transition diseases and supports our previous reports that revealed clear separation of healthy and diseased cows on the basis of blood plasma metabolites profile at 4 wks before the onset of clinical signs of periparturient diseases in dairy cattle [49, 50].

## Conclusions

In conclusion, to the best of our knowledge this is the first study to relate concentrations of IL-1, IL-6, TNF, SAA, and lactate in the serum of would-be-RP cows with occurrence of RP starting at −8 wks prior to parturition and during the entire dry off period and during disease diagnosis. Elevated concentrations of several serum innate immunity variables and lactate reflect pathophysiological events occurring in would-be-RP cows prior to occurrence of disease. Since cytokines and APPs measured are part of the innate immunity, which is a general and non-specific immune response, they cannot be used to identify specific diseases; however, their utilization in identification of cows that are susceptible to periarturient diseases is of great value.

## Abbreviations

APPs: acute phase proteins
APR: acute phase response
AUC: area under the curve
BCS: body condition score
BHBA: β-hydroxybutyric acid
CON: control/healthy cows
CP: crude protein
CV: coefficients of variation
DMI: dry matter intake
ELISA: enzyme-linked immunosorbent assay
Hp: haptoglobin
Hs: health status
IL: interleukin
LSM: least-squares means
MUN: milk urea nitrogen
NEB: negative energy balance
NEFA: non-esterified fatty acids
PCA: principal component analysis
PLS-DA: partial least square - discriminate analysis
ROC: receiver-operator characteristic
RP: retain placenta
SAA: serum amyloid A
SCC: somatic cell counts
SEM: standard error of the mean
TNF: tumor necrosis factor
TS: total solids
VIP: variable importance in the projection

## References

[1] Kimura K, Goff JP, Kehrli ME, Reinhardt TA (2002). Decreased neutrophil function as a cause of retain placenta in dairy cattle. J Dairy Sci..

[2] Roberts SJ (1986). Purpereal infections, uterine infections and diseases. Veterinary obstetrics and genital diseases.

[3] Ametaj BN, Zebeli Q, Iqbal S. Nutrition, microbiota and endotoxin- related diseases in dairy cows. Revista Brasileira de Zootecnia. 2010;39:433-444. On-line version ISSN 1806-9290.

[4] McNaughton AP, Murray RD (2009). Structure and function of the bovine fetomaternal unit in relation to the causes of retained fetal membranes. Vet Record..

[5] LeBlanc SJ (2008). Postpartum uterine disease and dairy herd reproductive performance - A review. Vet J..

[6] Bertoni G, Trevisi E (2013). Use of the liver activity index and other metabolic variables in the assessment of metabolic health in dairy herds. Vet Clin Food Anim..

[7] Zebeli Q, Sivaraman S, Dunn SM, Ametaj BN (2011). Interminent parenteral administration of endotoxin triggers metabolic and immunological alterations typically associated with displaced abomasum and retain placenta in periparturient dairy cows. J Dairy Sci..

[8] Lazlo K, Otto S, Viktor J, Laszlone T, Beckers JF, Endre B (2009). Examination of some reproductive indices of peripartal period in relation with energy metabolism in dairy cows. Magyar Allatorvosok Lapja..

[9] Ospina PA, Nydam DV, Stokol T, Overton TR (2010). Evaluation of nonesterified fatty acids and beta-hydroxybutyrate in transition dairy cattle in the northeastern United Stated: Critical thresholds for prediction of clinical diseases. J Dairy Sci..

[10] Seifi HA, Dalir B, Farzaneh N, Mohr M, Gorji- Dooz M (2007). Metabolic changes in cows with or withour retain fetal membranes in transition period. J Vet Med.

[11] Quiroz-Rocha GF, LeBlanc S, Duffield T, Wood D, Leslie KE, Jocobs RM (2009). Evaluation of prepartum serum cholesterol and fatty acids concentrations as predictors of postpartum retention of the placenta in dairy cows. J Am Vet Med Assoc..

[12] Van Werven T, Schukken YH, Lloyd J, Brand A, Heeringa T, Shea M (1992). The effects of duration of retain placenta on reproduction, milk production, postpartum disease and culling rate. Theriogenology..

[13] Esslemont RJ, Kossaibati MA (1996). Incidence of production diseases and other health problems in a group of dairy herds in England. Vet Rec..

[14] Coleman DA, Thane WV, Dailey RA (1985). Fctors affecting reproductive performance of dairy cows. J Dairy Sci..

[15] Huzzey JM, Duffield TF, LeBlanc SJ, Veira DM, Weary DM, von Keyserlink MAG (2009). Short communication: Haptablobin as an early indicators of metritis. J Dairy Sci..

[16] Lucey S, Rowlands GJ, Russel AM (1986). Short- term association between disease and milk yield of diary cows. J Dairy Res..

[17] Guard C. Set up fresh and milking cows for successful A.I. Hoard’s Dairy man. 1999. p. 8–9.

[18] Canadian Council on Animal Care (1993). Guide to the Care and Use of Experimental Animals.

[19] NRC guidelines (2001). Nutrient requirements of dairy cattle.

[20] Ametaj BN, Koenign KM, Dunn SM, Yang WZ, Zebeli Q, Beauchemin KA (2009). Backgrounding and finishing diets are associated with inflammatory responses in feedlot steers. J Anim Sci..

[21] Iqbal S, Zebeli Q, Mazzolari A, Dunn SM, Ametaj BN (2012). Barley grain- based diet treated with lactic acid and heat modulated plasma metabolites and acute phase response in dairy cows. J Anim Sci..

[22] Iqbal S, Zebeli Q, Mazzolari A, Dunn SM, Ametaj BN (2010). Feeding rolled barley grain steeped in lactic acid modulated energy status and innate immunity in dairy cows. J Dairy Sci..

[23] Xia JG, Psychogios N, Young N, Wishart DS (2009). MetaboAnalyst: a web server for metabolomic data analysis and interpretation. Nucleic Acids Res..

[24] Xia J, Sinelnikov IV, Han B, Wishart DS. MetaboAnalyst 3.0––making metabolomics more meaningful. Nucleic Acids Res. 2015. doi: 10.1093/nar/gkv380. SAS Institute Inc., Cary, NC, USA, Version 9.2. 10.1093/nar/gkv380 PMC4489235 25897128

[25] Janeway CA, Travers P Jr, Walport M and Shlomchik MJ. Imunobiology. The immune system in health and disease 5th edition. New York: Garland Science. 2001. ISBN-1D 0-8153-3642-X. (Chapter 2, pp).

[26] Islam R, Kumar H, Nandi S, Rai RB (2013). Determination of aniinflammatry cytokine in periparturient cows for prediction of postpartum reproductive health. Theriogenology..

[27] Boby J, Kumar H, Ganesan A, Singh SK, Narayanan K (2013). Short communication: Pre- partum serum cytokine levels as a potential tool for the prediction of retention of fetal membranes in cross- bred cows. Advances in Anim and Vet Sci..

[28] Trevisi E, Jahan N, Bertoni G, Ferrari A, Minuti A (2015). Pro-inflammatory cytokine profile in dairy cows: consequences for new lactation. Ital J Anim Sci..

[29] Sheldon IM, Noakes DE, Rycroft AN, Dobson H (2001). Acute phase protein responses to uterine bacterial contamination in cattle after calving. Vet Rec..

[30] Ametaj BN, Hosseini A, Odhiambo JF, Iqbal S, Deng Q, Lam TH, Farooq U, Zebeli Q, Dunn SM. Application of acute phase proteins for monitoring inflammatory states in cattle. In: Francisco Veas, editors. Acute Phase Proteins as Early non-specific Biomarkers of Human and Veterinary Diseases. Rijeka, Croatia: InTech; 2011. pp. 299–354.

[31] Ametaj BN, Bradford BJ, Bobe G, Nafikov RA, Lu Y, Young JW, Beitz DC (2005). Strong relationship between mediators of the acute phase response and fatty liver in dairy cows. Can J Anim Sci..

[32] Wassell J (2000). Haptoglobin: function and polymorphism. Clin. Lab..

[33] Loor JJ, Dann HM, Guretzky NAJ, Everts RE, Oliviera R, Green CA, Litherland NB, Rodriguez- Zas SL, Lewin HA, Drackley JK (2006). Plane of nutrition prepatrum alters hepatic gene expression and function in dairy cows as assessed by longitudinal transcript and metabolic profiling. Physiol Genomics..

[34] Bionaz M, Trevisi E, Calamari L, Librandi F, Ferrari A, Bertoni G (2007). Plasma paraoxanase, health, inflammatory conditions, and liver function in transition dairy cows. J Anim Sci..

[35] Steel JH, O'Donoghue K, Kennea NL, Sullivan MHF, Edwards A (2005). Maternal origin of inflammatory leukocytes in preterm fetal membranes, shown by fluorescence in situ hybridisation. Placenta..

[36] Drackley JK (1999). Biology of dairy cows during the transition period: The final frontier?. J Dairy Sci..

[37] Bundgaard H, Kjeldsen K, Suarez KK, van Hall G, Simonsen L, Qvist J, Hansen CM, Moller K, Fonsmark L, Lav Madsen P, Klarlund PB (2003). Endotoxemia stimulates skeletal muscle Na + -K + -ATPase and raise bood lactate under aerobic conditions in humans. Am J Physiol Heart Circ Physiol..

[38] Davis SR, Farr VC, Prosser CG, Nicholas GD, Turner SA, Lee J, Hart AL (2004). Milk L-lactate concentration is increased during mastitis. J Dairy Sci..

[39] Zhang G, Hailemariam DM, Dervishi E, Deng Q, Goldansaz A, Dunn SM, Ametaj BN. Activation of innate immunity in transition dairy cows before clinical appearance of milk fever. ADSA-SAS-CSAS Joint Annual Meeting. 2014.

[40] Zhang G, Hailemariam D, Dervishi E, Deng Q, Goldansaz A, Dunn SM, Ametaj BN (2015). Alterations of innate immunity reactants in transition dairy cows before clinical signs of lameness. Animals..

[41] Husain Z, Huang Y, Seth P, Sukhatme V (2013). Tumor-derived lactate modifies antitumor immune response: Effect on myeloid-derived suppressor cells and nk cells. J Immunology.

[42] Fischer D, Li Y, Ahlemeyer B, Krieglstein J, Kissel T (2003). In vitro cytotoxicity testing of polycations: influence of polymer structure on cell viability and hemolysis. Biomaterials..

[43] Gottfried E, Kunz-Schughart LA, Ebner S, Mueller-Klieser W, Hoves S, Andreesen R, Mackensen A, Kreutz M (2006). Tumor-derived lactic acid modulates dendritic cell activation and antigen expression. Blood..

[44] Shime H, Yabu M, Akazawa T, Kodama K, Matsumoto M, Seya T, Inoue N (2008). Tumor-Secreted Lactic Acid Promotes IL-23/IL-17 Proinflammatory Pathway. J Immunology..

[45] Ingvarsten KL, Andersen JB (2000). Integration of metabolism and intake regulation: a review focusing on periparturient animals. J Diary Sci..

[46] Sartori R, Pontes GCS, Monteiro PJL, Nascimento AB, Melo LF, Wiltbank MC (2013). 105 retained fetal membranes: incidence and effect on milk production and reproductive performance in dairy cows. Rep Fert Development..

[47] Theas MS, De Laurentiis A, Lasaga M, Pisera D, Duvilanski BH, Seilicovich A (1998). Effect of lipopolysaccharide on tumor necrosis factor and prolactin release from rat anterior pituitary cells. Endocrine..

[48] Kushibiki S, Hodate K, Shingu H, Obara Y, Touno E, Shinoda M, Yokomizo Y (2003). Metabolic and lactational responses during recombinant bovine tumor necrosis factor-α treatment in lactating cows. J Dairy Sci..

[49] Hailemariam D, Mandal R, Saleem F, Dunn SM, Wishart DS, Ametaj BN (2014). Identification of predictive biomarkers of disease state in transition dairy cows. J. Dairy Sci..

[50] Hailemariam D, Mandal R, Saleem F, Dunn SM, Wishart DS, Ametaj BN (2014). Metabolomics approach reveals altered plasma amino acid and sphingolipid profiles associated with pathological states in transition dairy cows. Current Metabolomics..

